# Health behaviour change of people living with HIV after a comprehensive community-based HIV stigma reduction intervention in North-West Province in South Africa

**DOI:** 10.1080/17290376.2014.985700

**Published:** 2014-12-12

**Authors:** H. Christa Chidrawi, Minrie Greeff, Q. Michael Temane

**Affiliations:** ^a^ PhD (Psychiatric Nursing), is a candidate in the Africa Unit for Transdisciplinary Health Research, North-West University, Potchefstroom Campus, Private Bag x6001, Potchefstroom2520, South Africa; ^b^ PhD (Psychiatric Nursing), is a professor in research in the Africa Unit for Transdisciplinary Health Research, North-West University, Potchefstroom Campus, Private Bag x6001, Potchefstroom2520, South Africa; ^c^ Ph.D (Psychology), is a researcher in the Africa Unit for Transdisciplinary Health Research, North-West University, Potchefstroom Campus, Private Bag x6001, Potchefstroom2520, South Africa

**Keywords:** HIV stigma, stigma experiences, health behaviour, health behaviour change, stigmatisation liée au VIH, expériences de stigmatisation, comportement de

## Abstract

All over the world, health behaviour is considered a complex, far reaching and powerful phenomenon. People's lives are influenced by their own or others' health behaviour on a daily basis. Whether it has to do with smoking, drinking, pollution, global warming or HIV management, it touches lives and it challenges personal and community responses. Health behaviour, and health behaviour change, probably holds the key to many a person's immediate or prolonged life or death outcomes. The same can be said about communities, culture groups and nations. This SANPAD-funded study focused on research questions relating to health behaviour change for people living with HIV (PLWH) in the North-West Province in South Africa. It investigated whether a comprehensive community-based HIV stigma reduction intervention caused health behaviour change in PLWH. An quantitative single system research design with one pre- and four repetitive post-tests utilizing purposive sampling was used to test change-over-time in the health behaviour of 18 PLWH. The results of the study indicated statistical and/or practical significant change-over-time. The intervention not only addressed the health behaviour of PLWH, but also their HIV stigma experiences, HIV signs and symptoms and their quality of life in the context of being HIV positive. The recommendations include popularization of the comprehensive community-based HIV stigma reduction intervention and extending it to include a second intervention to strengthen health behaviour and quality of life for PLWH in the community at large.

## Résumé

La stigmatisation liée au VIH entrave l'accès aux soins et traitements, et accroit la transmission du VIH mondialement. La crainte de la stigmatisation empêche les personnes vivant avec le VIH (PVVIH) de saisir les opportunités d'accès aux soins de santé et de bénéficier des traitements. Elle affecte l'ensemble de la qualité de vie des PVVIH et leurs comportements de santé. Cette étude cherche ainsi à savoir si une intervention communautaire globale de réduction de la stigmatisation liée au VIH changerait le comportement de santé des PVVIH. L'objectif était d’étudier et tester l’évolution dans le temps des expériences de stigmatisation liée au VIH et des comportements de santé des PVVIH après l'intervention. Pour tester l’évolution dans le temps du comportement de santé de 18 PVVIH, on a utilisé une méthode de recherche expérimentale quantitative à système unique avec un test préalable, quatre tests postérieurs répétés, et un échantillonnage dirigé. Les mesures incluaient l'utilisation d'un questionnaire démographique, de l'Instrument sur la stigmatisation perçue par les PVVIH, de la Mesure révisée ACTG d'auto-rapport d'adhérence au traitement, des Stratégies révisées d'autogestion des symptômes comme mesure des comportements de santé, ainsi que de l'Instrument sur la qualité de vie orientée sur le VIH. Les assistants à la recherche ont été formés à employer ces instruments dans un contexte privé, pratique pour les participants. 18 PVVIH ont participé volontairement à la mesure de la réduction de la stigmatisation pendant un an après l'intervention et les résultats ont montré que la réduction de la stigmatisation liée au VIH entraîne un changement des comportements de santé, des symptômes du VIH moins intenses, l'amélioration de l'adhérence au traitement, une gestion responsable de la divulgation du VIH, l'amélioration des relations et de la qualité de vie. Il est recommandé d’étendre cette intervention à d'autres cultures, provinces et communautés vulnérables urbaines ou rurales. Les mécanismes pour maintenir la participation active des PVVIH à la réduction communautaire de la stigmatisation liée au VIH méritent d’être étudiés et devraient générer des structures de soutien communautaire des participants des futures interventions.

## Introduction

This study forms part of a large SANPAD project dealing with the stigma experiences of people living with HIV (PLWH) and the stigmatization by people living close (PLC) to them. HIV remains a highly stigmatized illness throughout the world and the focus, in this article, is on stigma and the health behaviour of the PLWH. Not only is HIV complex in nature but HIV stigma impacts on the physical, psychological, social and financial dimensions of PLWH, as it violates general health, basic human rights, social and economic freedom and quality of life (Chirwa, Greeff, Kohi, Naidoo, Makoae & Dlamini, *et al.* 2009; Dlamini, Wantland, Makoae, Chirwa, Kohi & Greeff, *et al.* 2009). Holzemer, Uys, Makoae, Stewart, Phetlhu, Dlamini, *et al.* (2007) conceptualized types of HIV stigma as internal stigma, received stigma and associated stigma. Other authors refer to self-stigma (Mak, Cheung, Law, Woo, Li & Chung 2007), perceived stigma (Link, Yang, Phelan & Collins 2004) and secondary stigma (Ogden & Nyblade 2005). These, in turn, lead to life-intrusive consequences, such as decreased social participation, personal and emotional frailty and diminished physical and mental health (Asiedu 2010; Cahill & Valadez 2013; Rensen, Bandyopadhyay, Gopal & Van Brakel 2011), and thus also have a serious effect on the health behaviour of PLWH. The effects of HIV stigma often become an added burden to existing compromised health behaviour of PLWH rendering them vulnerable to challenges of social participation, self-responsibility and behaviour modification as well as choices regarding their sexual partners, lifestyle and medical care (Genberg, Kawichai, Chingono, Senday, Chariyalertsak, Konda, *et al.* 2008; Mallory, Johnson, Neilands, Dilworth, Morin, Chesney, *et al.* 2007; Taylor 2001; Zimmerman, Olsen & Bosworth 2000). The interplay between HIV stigma and HIV health behaviour is of critical concern in defeating the AIDS pandemic and restoring quality of life for PLWH (Bhagwanjee, Petersen, Akintola & George 2008; Greeff, Uys, Wantland, Makoae, Chirwa & Dlamini, *et al.* 2010). Health behaviour is as dynamic and complex as any other behaviour and always has meaning and purpose (Bonell & Imrie 2001). It sometimes resides in dimensions of character or attitude, will power, physical/psychological/mental strengths and resilience or even peer pressure, psychosocial interaction or one's spiritual relationship with a higher being (Setswe 2009). In some African contexts, for instance, health or ill health is seen as a consequence of human behaviour, but it can also be sanctioned by God or brought on by the ancestors (Pinkoane 2005), which complicates straightforward motivation towards behaviour change.

This suggests that potential health behaviour change interventions have to be comprehensive, cover a broad spectrum of challenges and achieve fundamental shifts in attitude, belief systems and power relations (Duncan, Harrison, Toldson, Malaka & Sithole 2005; Link & Phelan 2001). It seems like innovative efforts surpassing the boundaries of single disciplines, and moving into lesser known territories of transdisciplinarity, could assist the behavioural sciences of the day to reduce risky health behaviour and instill ‘good’ health behaviour (Jordan & Bazzarre 2002; Kelly, Murphy, Sikkema & Kalichman 1993) in the face of HIV stigma. A number of conventional HIV stigma-related behaviour modification mechanisms as well as contemporary health behaviour change models exist in the literature.

### Conventional behaviour change mechanisms

*Acceptance of HIV as a lifetime illness* requires the courage to manage HIV stigma and refusal to allow such stigma to become a hidden and continuous burden or secret diagnosis (Weiss, Ramakrishna & Somma 2006). Many patients in the USA are now viewing and managing HIV as just another manageable chronic disease packaged with modified behaviour and a lifelong treatment regime like diabetes or hypertension (Holzemer 2012; Mbonye, Nakamanya, Birungi, King, Seeley & Jaffar 2013).

*Increased information and education* leads to deeper understanding of HIV stigma (Bonell & Imrie 2001) and a lack of knowledge in PLWH can lead to being enveloped in a personal cloud of stigma and not realizing the manageability of the illness if treated correctly (Allick 2012). Education, information sharing, counselling and shared advice could remedy health behaviour driven by fears and unwillingness (Greeff, Phetlhu, Makoae, Dlamini, Holzemer, Naidoo, *et al.* 2008). A dynamic HIV workplace programme of a multinational brewing company reported that in spite of a positive response sparked by educational and promotional activities only 15–32% employees and their spouses in 5 African countries participated in HIV counselling and testing (HCT) programmes offered by the company (Van der Borght, Schim van der Loeff, Clevenberg, Kabarega, Kamo, Van Cranenburgh, *et al.* 2010).

*HCT* probably remains one of the more important health behaviour expected to stem the tide of HIV and is therefore included in the South African National Strategic Plan (NSP, 2012–2016) (SANAC 2011). One of the advantages is a sense of relief in knowing your HIV status and opens the subsequent potential to responsibly re-plan and modify one's lifestyle, risky behaviour, sexual partners, condom use, abstinence, treatment and supportive friendships (Bonell & Imrie 2001). HIV stigma and the fear of being identified, unfortunately, remains a critical challenge to HIV testing, care and prevention (Florom-Smith & De Santis 2012).

*Access and adherence to treatment* is as important as HCT and the barriers to making the decision and taking treatment action is often HIV stigma related (Allick 2012). Non-uptake of treatment behaviour is pretty much consequential to HIV stigma and its challenges UNAIDS 2004). Florom-Smith and De Santis (2012) commented from a synthesized literature review that experiencing stigma often cause PLWH to fail adherence to their prescribed treatment regimens. This study found that the main reason offered for missing dosages of medication was having to take the medicine at a specific time where others might notice and ask questions about the medication.

*Responsible HIV disclosure management remains complex* and the disclosure decision should always be well informed as the disclosing PLWH have no control over reactions of other people afterwards, the subsequent disclosure by third parties or the potential consequences after any incident of disclosure. This means that there is anxiety attached to every disclosure of one's HIV status as the response can never be predicted (Greeff 2013). One of the reasons for disclosure is responsible negotiation of sexual partners about condom-protected sex or consenting unprotected sex or one-partner-only relationships (Bonell & Imrie 2001). It furthermore seems evident that disclosure is always vulnerable where HIV stigma is high and Sales, Ryan, Silver, Sarkisian and Cunningham (2007) report that stigmatized PLWH avoided all medical care, even emergency treatment, for the fear of disclosure of their serostatus which they believed would lead to inferior care and being treated as though they were a danger to staff. If stigma is prevalent, PLWH would often have conflict over whether to disclose their serostatus and from whom to seek support, if at all (Florum-Smith & De Santis 2012).

*Support-seeking behaviour* is a human response in the face of adversity such as stigmatization, discrimination, job insecurity, a decreased quality of life and a need for compassion, care, acceptance, openness, friendship, sharing and respect from significant others (Relf, Mallinson, Pawlowski, Dolan & Dekker 2005). Support groups have traditionally been recognized for their value in providing emotional support, lessening the burden of stigma, exploring new ideas on how to cope and Foster and Gaskins (2009) identified spiritual sharing as supportive behaviour for PLWH.

### Contemporary health behaviour change models, theories and interventions

Behaviour change interventions for HIV stigma reduction require a design that accurately speaks of specific outcomes, needs and contexts (Funnel & Rogers 2011). There seems to be a paucity in research findings on behaviour change that offers exhaustive answers to the challenges of HIV stigma (Moore & Charvat 2007). Strangle Lloyd, Brady, Holland and Baral (2013) recently emphasized the critical need for the identification of effective interventions for stigma reduction and lessening of discrimination on a bigger scale. Zimmerman *et al.* (2000) refer to the ‘stages of change’ model for gradual change in behaviour. The person moves from being disinterested, unaware or unwilling to the act of activating change. First, a form of pre-contemplation, then consideration of a change (contemplation), and then taking deliberate action to make a behaviour change as meant as the beginning of a lifelong change (Norcross, Krebs & Prochaska 2011). This trans-theoretical model underpins a number of other behaviour change theories, such as the BASNEF model with locus of control theory (Hubley 1988), health belief model (Stretcher & Rosenstock 1997), motivational interviewing (Zimmerman *et al.* 2000), cognitive-behavioural therapy (Heijnders & Van der Meij 2006) and the well-known applied 12-step programmes to change co-dependent behaviour (Zimmerman *et al.* 2000).

The principles and elements of all the above theories or models can guide change processes. PLWH are not powerless and can, in different circumstances, become effective change agents within their own skin, circumstance and culture (Gladwell 2002). Mashadi (2012) suggests that individuals utilize voluntary cognitive behaviour strategies, such as positive goal setting, the identification of personal strengths and scheduled distraction activities, to overcome negativity or depression and to direct determined new behaviour, like changed sexual practices. CliffsNotes.com (2013) provide five types of hypotheses that would reduce prejudice and therefore be useful for HIV stigma-related behaviour change. They state that (a) an appropriate education and higher self-esteem will reduce prejudice; (b) prejudice will decline if differing groups spend quality time together; (c) prejudice will decline if conflicting groups work together for a communal goal; (d) discriminative behaviour should be outlawed and (e) collaborative interactions between differing groups will reduce prejudice. Various other researchers further contributed to the field of HIV stigma-related behaviour change. Corrigan (2004) suggested that changing approaches in terms of public stigma should be grouped into three categories; namely (a) protesting prejudice, (b) education and (c) personal contact with PLWH. Kendra, Cattaneo and Mohr (2012) and Boyd, Katz, Link and Phelan (2010) concurred on matters of education and contact with PLWH. Boyd *et al.* (2010) also emphasized that personal contact with the stigmatized could lessen anger, blame, social distancing and fear. Kendra *et al.* (2012) reiterated education as a means of reducing HIV stigma and found traditional lecturing was less effective than personal contact and experiential learning techniques.

An innovative approach to health-related behaviour change in the future could thus stem from conventional contributions and move beyond the borders of separate disciplines to cut across conventional scope and practice (Funnel & Rogers 2011). Emerging trans-disciplinarity and trans-disciplined partnerships can facilitate HIV stigma and health-related behaviour change (Nicolescu 2007) and be inspired by a broad perspective of diversity, respectful confrontation and mutual transformation of disciplines. This will require scientifically transformed minds that would strive for synergy and collective encounter across academic barriers towards the greater good (Selcer 2011) of changing HIV stigma behaviour.

## Problem statement

HIV stigma and stigmatization impede access to care and treatment and fuel HIV transmission all over the world. Fear of stigma or stigmatization causes people to ignore opportunities to learn about HIV, get to know their HIV status, utilize health-care facilities and make use of treatment. Stigma lets people maintain potentially risky sexual relations for the fear of being questioned should they change. PLWH are often fearful to disclose their HIV status and thus compromise supportive relationships and quality of life. The more PLWH are exposed to HIV stigma experiences, the more difficult it becomes to face their challenges and take responsibility to break the silence, and to modify their health habits and lifestyle choices. It seems critical to invest in HIV stigma reduction and limit its crippling effects on the health behaviour of PLWH.

The *research question* core to this study was whether the comprehensive community-based HIV stigma reduction intervention will result in change and *the research objective* therefore was to observe change in the HIV stigma experiences and health behaviour of PLWH after a comprehensive community-based HIV stigma reduction intervention. A quantitative single-system design (De Vos, Strydom, Fouche & Delport 2005) with a pre- and four repetitive post-tests measures (01×02 03 04 05) was conducted in both an urban and a rural setting.

## Research method

*The sample* of the study consisted of PLWH in both an urban (Potchefstroom) and a rural (Ganyesa) district of the North-West Province, South Africa. Existing community public benefit organizations, HIV health-care clinics and mediators (who had a trusting relationship with PLWH) assisted with purposive voluntary sampling. The identified PLWH had to be over 18 years of age and conversant in either Afrikaans, English or Setswana. They had to be HIV positive for at least six months prior to their inclusion. The sample of 18 PLWH was relatively small due to the therapeutic nature of the intervention in the bigger study and the intended interactive contact between PLWH and PLC to them during the workshops.

Ten PLWH (9 females and 1 male) were from the Potchefstroom urban district and eight PLWH (5 females and 3 males) from the rural district, Ganyesa, in the North-West Province of South Africa. PLWH were required to sign forms confirming their informed consent for participation and for recording processes (see ethical considerations). They were included in a preparatory workshop relating to possible disclosure of HIV status during the intervention and the schedule of one pre- and four post-tests measurements.

*The intervention* for this study was adapted from the validated intervention manual by Uys Chirwa, Kohi, Greeff, Naidoo, Makoae, *et al.* (2009). It was based on three tenets, namely (a) information sharing on HIV stigma and how to cope with it, (b) equalizing of relationships between PLWH and PLC to them through increased interaction and contact and (c) empowerment to implement a HIV stigma reduction project in their communities.

The comprehensive community-based HIV stigma reduction intervention consisted of mainly three activities. There was first a two-day lecture and activity-based workshop for PLWH in both urban and rural settings, which focused on their personal understanding of HIV stigma, responsible disclosure management and personal strengths identification. This initial workshop was then followed by a series of six three-day workshops attended by all PLWH and particular PLC to them from six designated groupings. These refer to, first a group of their spouses/partners, followed by a group of a child over 15 for each PLWH, then groups of a family member, a friend, a spiritual leader and finally a community member for all PLWH. The first day of these activity-based group-workshops for PLWH and people close to them focused on an understanding of HIV stigma and coping with it, as well as the relationships among them. All the workshops had two facilitators (one HIV infected and one non-HIV infected person) in each group. The second day focused on learning how to plan a project for HIV stigma reduction in their own community. It offered each of the designated groupings an opportunity to plan their own project for community members of similar designated categories to their own, e.g. Partners with partners. They had a four-week period to implement their projects and were facilitated by the researchers. This meant that there were six projects running in Potchefstroom (urban setting) and six projects in Ganyesa (rural setting) simultaneously. On the third and last day of the intervention, the different groupings personally presented their project reports to invited community members and the research team. Each project was evaluated and small prizes were presented to the most outstanding urban and rural community HIV stigma reduction projects.

*The data collection* for health behaviour of PLWH included a demographic questionnaire utilized pre-intervention alongside four valid and reliable measuring instruments for the pre-test and four repetitive (three-monthly) post-test measures. These were the Validation of HIV/AIDS stigma instrument for PLWA (HASI-P), Revised ACTG Self-report Adherence to Medication measure, the Revised Self-care Symptom Management strategies (SSC-HIVrev) and The HIV/AIDS-targeted Quality of Life instrument (HAT-QoL).

### Description of the measuring instruments

*A Demographic questionnaire* that included personal data, such as age, gender, marital status, number of children, education level and employment status was utilized (Greeff, Uys, Wantland, Makoae, Chirwa, Dlamini, *et al.* 2010).

*Revised ACTG self-report adherence to medication measure* stems from the AIDS Clinical Trials Group's instrument (ACTG-Rev) that initially consisted of 14 self-reported reasons for missing medications. In 2006, Holzemer and colleagues reported on an additional factor analysis conducted to reduce the ACTG-Rev to a 9-item instrument with a one-factor solution and two scores that are calculated from the revised 9-item scale as a measure of health behaviour. Respondents rate how often in the past month they have missed their ARV medications for a particular reason, on a scale of 1–4 (never too often). The resulting score ranges from 9 to 36, where higher scores mean that the person missed more doses (Holzemer, Hudson, Kirksey, Hamilton & Bakken 2001).

*The Validation of HASI-P* is a 33-item instrument developed by Holzemer *et al.* (2007). The instrument reflects a total score of perceived HIV stigma and the mean scores of how PLWH experienced each of six HIV and AIDS stigma dimensions (verbal abuse (VA), negative self-perception (NSP), health-care neglect (HCN), social isolation (SI), fear of contagion (FC) and workplace stigma). It was initially validated by Holzemer *et al.* (2007) with a sample of 1477 respondents from five African countries where a Cronbach reliability coefficient of 0.94 was found.

*The Revised self-care symptom management strategies (SSC-HIVrev)* is a measure of health behaviour described by Holzemer *et al.* (2001) and measures the intensity and frequency of 72 common signs and symptoms of HIV. Part one of the instrument features 45 HIV-related physical and psychological symptoms clustered according to 11 factor scores. The initial reliability ranged between 0.76 and 0.91. Part two has 19 HIV-related symptoms that do not cluster into factors but offer clinical knowledge. These 64 items were used while the eight gynaecological symptoms for women, in part three, were not utilized for this study. The HIV Sign and Symptom check-List (rev.) has been used extensively in Southern Africa with a Cronbach reliability estimate of 0.96 for the total scale (Dlamini *et al.* 2009).

*The HIV/AIDS-targeted quality of life instrument (HAT-QoL)* as developed by Holmes and Shea (1998) is a 34-item HIV-specific instrument to identify quality-of-life concerns of PLWH. The scale measures nine dimensions: overall function, life satisfaction (LS), health worries (HW), financial worries (FW), medication worries (MW), HIV mastery, disclosure worries (DW), provider trust and sexual function (SF). The subscales are scored on a Likert scale rating of 1 (none of the time) to 5 (all the time) during the past four weeks. The final score is transformed into a linear 0–100 scale where 100 is the best score possible. The Cronbach alpha reliability coefficient was 0.86 at baseline, and 0.96 and 0.95 for two subsequent assessments indicating excellent consistency reliability of the items (Dlamini *et al.* 2009; Holmes & Shea 1998).

Research assistants were trained two weeks before the intervention as preparation for them to execute data collection and facilitate the workshops. They then received the names of participants from the mediators, scheduled appointments with them and ensured transport and logistical comfort for the commencement of the intervention at the North-West University campus.

## Data analysis

The Statistical Package for the Social Sciences (SPSS, version 21) for Windows was utilized for data analysis. Descriptive statistics including mean, mean square error and p values were computed. Effect sizes calculated the practical significance of mean differences for changes across time. The analysis took the dependency on data collected over time relating to specific persons into account (McCoach 2010). The urban–rural data were pooled for analysis and included aspects of statistical as well as practical significance.

## Ethical considerations

Permission for this SANPAD-funded research component was obtained from the School of Nursing Science as well as from the North-West University Ethics Committee (NWU-OOO 11-09-A1) (30/03/2009-29/03/2014). The North-West Provincial Department of Health granted permission for the research. Basic ethical principles such as respect for human subjects, benevolence and justice were maintained (Burns & Grove 2009). *Respect* for participants was demonstrated by offering information about the study, criteria for their inclusion, the schedule for workshops and three-monthly measuring and their freedom to withdraw from the process at any time. It was also explained that anonymity was based on a coding system not linking their true identity with their specific responses at any time. There was only partial confidentiality since the participants worked together in groups. The participants signed a consent form if they were satisfied with the information and the intentions of the research team. *Benevolence* for participants was plentiful in the sharing of knowledge on HIV stigma and coping with it, identification of personal strengths, learning about responsible disclosure management, potential enhancement of close relationships and being exposed to project skills training. The *justice* for participants related to fair treatment and the management of possible risks by providing a safe environment, transport and a light lunch.

## Findings and discussion

The urban–rural data were pooled for analysis because there were no significant differences found between the two groups over time.

*The demographic and background information* of the 18 PLWH participants showed that the ages of participants ranged between 27 and 52 with an average age of 37 years. There were only three of the participants presently married, one widowed, two divorced and twelve were never married. Three participants had no children, six had one child, six other had two children and two had three children each. In terms of schooling, 7 passed grade 10, 2 passed grade 11 and 3 passed grade 12. None of the participants achieved a degree, 1 had a diploma, 7 a post-school certificate and 10 had no further education after school. There were seven employed, six lived on government grants, seven were supported by family and one earned a living by doing piece jobs. It seems like the PLWH were a middle-aged group with minimal education, general lack of employment and representative of the lower socio-economic population. The information from the demographic questionnaire, alongside data from the other instruments will form a collective basis for a thematic discussion of findings.

*The ARV treatment and adherence to treatment* of the group were measured by the ACTG questionnaire and combined with information from the demographic questionnaire. Although 3 of the 18 PLWH did not answer ARV questions in their demographic questionnaire, the ACTG gave a fairly comprehensive picture of the adherence to treatment of the PLWH. Table 1 combines the information of 18 PLWH (coded between 101 and 210) with nine reasons and four frequency categories for missing dosages of their ARV medication. This measure refers to the time-two measure directly after the intervention took place. Eight of the 18 people said that they have never forgotten to take their medicines while 7 gave reasons, and frequencies of using these reasons, for missing dosages of medication. Of these seven PLWH, five reported that they missed taking their medication four times or less over the last three months. The most frequent reason referred to difficulty to take medication at a specific time (Table 1). There were 2 of the 18 PLWH missing many dosages. The one person (coded 101) sometimes missed because of having to take it at specific times, being too busy, away from home, or oversleeping and often missed because s/he forgot. Person coded 106 marked all nine reasons for missed dosages with varying frequency of ratings. The behaviour scores on the ACTG scale for persons 101 and 106 were 20 and 26 respectively, while the ideal score is nine and the worst possible score is 36. Eleven of the 18 PLWH actually scored a perfect adherence score of nine. Responses on the demographic questionnaire indicated that one person who stopped ARVs once before because of a lack of regular food. Fourteen of the 18 PLWH reported diligent adherence to their treatment regimens with such reminders as an alarm clock, cell phone, daily radio programme or caring relative to ensure that they take their ARVs at the correct times. Where side effects of the ARVs were concerned, four of the PLWH indicated no trouble, four felt bloated, two experienced vomiting, two had skin rash and two reported pain because of the ARVs.

**Table 1.** PLWH reasons and frequency of using reasons for missing dosages of medication.

ACTG self-report on reasons and frequency of using these reasons for missing dosages of medication										
Reason	Avoid side-effects	Felt to sick	Too many pills	Felt depressed	Specific time impossible	Too busy	Away from home	Forgot	Slept	Behaviour 9–36
18 PLWH										
101	1	1	1	1	3	3	3	4	3	20
102	1	1	1	1	3	2	2	1	1	13
103	1	1	1	1	1	1	1	1	1	9
105	1	1	1	1	1	1	1	1	1	9
106	3	2	3	3	3	3	2	3	4	26
107	3	4	1	1	3	1	1	1	1	16
108	1	1	1	1	1	1	1	1	1	9
109	1	1	1	1	1	1	1	1	1	9
110	1	3	1	3	2	1	1	1	3	16
111	1	1	1	1	1	1	1	1	1	9
201	1	1	1	1	1	1	1	1	1	9
202	1	1	1	1	1	1	1	1	1	9
203	1	1	1	1	1	1	1	1	1	9
204	1	1	1	1	1	1	1	1	1	9
205	1	1	1	1	1	1	1	1	3	11
207	1	1	1	1	1	1	1	1	1	9
209	1	3	1	3	1	1	1	1	1	13
210	1	1	1	1	1	1	1	1	1	9

Note: never = 1, rarely = 2, sometimes = 3, often = 4 (behaviour score per coded participant – 9 better; 36 missed many).

These findings, where 16 of the 18 PLWH participating in the ACTG measure demonstrated strong adherence to their treatment regime after the start of the intervention, and 11 PLWH were exemplary in their adherence to treatment, imply that reasons for non-compliance should be addressed. The stronger reason for not taking medication directly relates to HIV stigma, as it links to a lack of privacy when taking medication at specific time slots.

*Disclosure of HIV status by PLWH* also featured in the demographic questionnaire. Nine of the 18 PLWH chose to disclose their HIV serostatus on the same day of their diagnosis. For the rest, three PLWH disclosed within the first week, four within the first two months and the last two only disclosed years after their diagnosis for the first time. In terms of first disclosure, nine PLWH disclosed to a family member, four disclosed to a partner, four to a friend and one to a neighbour. Subsequent disclosures by PLWH included family members and friends as priorities with fifteen and eleven PLWH respectively choosing these PLC, nine chose their spiritual leaders and eight disclosed to either a community member or a neighbour. Only seven PLWH reported that they subsequently disclosed to partners, six to one or more of their children and two disclosed their HIV status to a colleague.

This finding emphasizes the pertinence of the tenets underpinning this HIV stigma reduction intervention; one of which is the strengthening of relationships for the PLWH and PLC. As this will encourage people to share their HIV status, it will also encourage PLWH and PLC to live supportively towards each other. More findings supporting this aspect were found.

*Support by others towards PLWH* was questioned in the demographic questionnaire. It first asked by whom the PLWH were supported and second how were they supported. Family members, again, were the most preferred support system for 13 of the 18 PLWH. A partner, child or friend was chosen by seven, six and six PLWH, respectively. The mechanisms of support mentioned were encouragement, sharing worries and ‘being checked on’; each mentioned by three of the PLWH. Providing money, food, care and reminders to take medication were each mentioned by two PLWH. It seems like PLWH felt supported by all categories of PLC, even spiritual leaders and colleagues for some. Community members, as such, were not mentioned and that category was thus combined with the neighbour category.

Again, the importance of relationship building, a tenet underpinning this study, was emphasized. It will be important to keep this aspect alive in further HIV stigma reduction interventions (Table 2).

**Table 2.** PLWH disclosure of HIV status and their support system.

Disclosure behaviour of PLWH								
How long post HIV diagnosis?		Same day	2–5 days	2–3 weeks	1–2 months	1–3 years		
		9	3	2	2	2		
PLC categories	Partner	Child	Family member	Friend	Spiritual leader	Colleague	Neighbour	Nobody
PLWH disclosure to whom:								
First disclosure	4		9	4			1	1
Subsequent disclosure	7	6	15	11	9	2	8	1
PLWH existing support system	7	6	13	6	3	2	4	1

*A change in HIV stigma experiences of PLWH* as a result of the intervention was measured on the ‘Validation of the HIV/AIDS stigma instrument PLWA (HASI-P)’ for PLWH. Effect sizes were calculated for analysis of practical significance in cases where the limited number of PLWH offered little statistical power to indicate statistical significance. The information given in Table 3 reflects five different subscales of HIV stigma dimensions for the PLWH. The pre-intervention scores are given on time one. The comprehensive community-based HIV stigma reduction intervention occurred between times one and two. Thereafter, four three-monthly post-test scores followed on each subscale, to test possible change-over-time.

**Table 3.** PLWH experiences of dimensions of stigma over five times.

Dimensions	Mean scores							Effect sizes			
	Time 1	Time 2	Time 3	Time 4	Time 5	MSE	*p*	1 with 2	1 with 3	1 with 4	1 with 5
VA	12.1	9.8	8.2	8.2	8.3	1.25	0.1	2.06	3.49	3.49	3.4
NSP	7.39	6.8	6.19	6.69	6.32	4.76	0.26	0.27	0.55	0.32	0.49
HCN	7.6	7.1	7.04	7.1	6.97	0.03	0.03	2.89	3.23	2.89	3.64
SI	6.7	5.75	5.51	5.51	5.42	1.78	0.38	0.71	0.89	0.89	0.96
FC	7.7	6.63	6.31	6.42	6.08	0.34	0.03	1.84	2.38	2.2	2.78
**Total**	**43.61**	**37.94**	**35.01**	**35.47**	**34.69**	**12.61**	**0.02**	1.6	2.42	2.29	2.51

The subscale on VA is not statistically significant (*p* = .10) but the effect sizes between times one and two, one and three, one and four as well as one and five are all well larger than 0.5, indicating practical significance. The mean 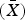 scores decreased from 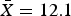 at time one to 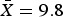 at time two and 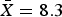 at time five. The NSP scores of the PLWH is not statistically significant (*p* = .26) either. Again, the effect sizes of the decrease between times one and three and times one and five indicate practical significance at 0.55 and 0.49. The HCN subscale indicates a statistically significant improvement in the HIV stigma experience of PLWH (*p* = .03). The four effect sizes between 2.89 and 3.64 are indicative of practical significance as well. The subscale of SI had effect sizes between 0.71 and 0.96 (>0.5) and thus showed practical significance and an improvement in the experiences of SI by PLWH. FC indicated statistical significance (*p* = .03). The total stigma score as calculated from the different subscales indicates a statistically significant (*p* = .02) improvement-over-time in the HIV stigma experiences of the PLWH, as well as a practical significance with effect sizes larger than 0.50.

The results on the HASI-P thus demonstrate a change in the HIV stigma experiences of PLWH following the comprehensive community-based HIV stigma reduction intervention. As these experiences have improved, and given the tenets of the intervention, PLWH felt more knowledgeable about HIV stigma and the ability to cope with it and to manage their physical HIV symptoms.

*The intensity and frequency of HIV signs and symptoms experienced by PLWH* were measured (over five times) on ‘The Revised self-care symptom management strategies (SSC-HIVrev) as measure of health behaviour’ (Holzemer *et al.* 2001). The scoring sheet allows choices of ‘do not have the problem, mild, moderate and severe’ in terms of the listed signs and symptoms. Table 4 indicates mean scores of all 18 PLWH. A low score (nine) reflects (do not have the problem) and a high score (36) thus reflects high intensity and frequency of a sign or symptom. Five of the nine subscales reflect a statistically significant decline of signs and symptoms in PLWH after the comprehensive community-based HIV stigma reduction intervention. These were fatigue (*p* = .05), numbness (*p* = .01), rectal itch (*p* = .03), bruising (*p* = .02) and gynaecological symptoms (gynael) (*p* =  <.01). Three of the remaining signs and symptoms, namely fear, gastro intestinal symptoms (GI_upset) and sore throat decreased with practical significance and effect sizes larger than 0.50. Especially the gastro intestinal symptoms decreased with effect sizes of 1.07 between times one and two directly after the intervention, and effect sizes of 2.47 between times one and three, 0.82 between times one and four and 1.66 between times one and five. The subscale and symptom with the least change-over-time after the intervention was shortness of breath (SOB). The times one and three effect size of 0.29 and one and five effect size of 0.4 indicate some practical significance in the decrease. The gynaecological scale was included for the female participants and reflected the highest intensity and frequency mean scores of all the signs and symptoms but also the largest effect sizes of improvement on top of the clear statistical significance.

**Table 4.** HIV signs and symptoms of PLWH over five times

PLWH – Stigma symptoms at five times								Effect sizes of each time with time 1			
Variables	Time 1	Time 2	Time 3	Time 4	Time 5	MSE	*p*	1 with 2	1 with 3	1 with 4	1 with 5
Fatigue	6.22	6.07	4.88	5.76	4.99	0.85	0.05	0.16	1.45	0.5	1.33
Fear	7.13	6.01	5.26	5.84	5.66	4.89	0.15	0.51	0.85	0.58	0.66
GI_upset	8.61	7.61	6.29	7.84	7.05	0.88	0.11	1.07	2.47	0.82	1.66
SOB	4.09	3.99	3.78	4	3.66	1.16	0.84	0.09	0.29	0.08	0.4
Sore throat	4.26	4.67	4.12	4.41	4.02	0.05	0.23	1.83	0.63	0.67	1.07
Numbness	6.57	5.44	4.49	4.77	4.95	0.12	0.01	3.26	6	5.2	4.68
Rectal_Itch	3.96	3.34	3.07	3.12	3.13	0.29	0.03	1.15	1.65	1.56	1.54
Bruising	5.09	4.61	4.22	4.1	4.29	0.33	0.02	0.84	1.51	1.72	1.39
Gynael	12.81	10.47	9.42	9.09	9.25	1.17	<0.01	2.16	3.13	3.44	3.29

The stigma reduction intervention seemed to have made a difference to the intensity and severity of experienced signs and symptoms of HIV. It could be that understanding HIV, HIV stigma and coping with HIV stigma better, being able to share these experiences with certain PLC and feeling supported in spite of being HIV positive and stigmatized, lightened the burden for PLWH. This was further observed in a focus on the quality of life of PLWH.

*Quality of life concerns for the PLWH* were assessed on ‘The HIV/AIDS-targeted quality of life instrument (HAT-QoL)’ (Holmes & Shea 1998) and summarized in Table 5.

**Table 5.** Quality of life concerns of PLWH over five times

PLWH – worrying factors over five times								Effect sizes of each time with time 1			
Variables	Time 1	Time 2	Time 3	Time 4	Time 5	MSE	*p*	1 with 2	1 with 3	1 with 4	1 with 5
HATQ_Total	86.38	85.89	92.95	89.5	85.83	116.05	0.52	0.05	0.61	0.29	0.05
HATQ_LS	86.09	91.39	83.37	89.55	89.11	119.64	0.66	0.48	0.25	0.32	0.28
HATQ_HW	77.39	85.45	91.21	92.05	82.24	137.13	0.01	0.69	1.18	1.25	0.41
HATQ_FW	32.46	40.2	40.3	36.57	43.22	23.38	0.45	1.6	1.62	0.85	2.23
HATQ_MW	90.01	94.02	96.97	93.8	95.92	91.44	0.45	0.42	0.73	0.4	0.62
HATQ_DW	77.39	89.96	95.36	91.9	89.54	17.48	0.02	3.01	4.3	3.47	2.91
HATQ_PT	83.77	75.72	87.88	88.66	79.1	91.03	0.26	0.84	0.43	0.51	0.49
HATQ_SF	66.09	78.04	84.12	90.64	84.89	37.94	0.08	1.94	2.93	3.99	3.05

The HATQ_Total refers to six overall concerns during the four weeks prior to measurement and included satisfaction with physical activity or being limited by physical ability, pain, tiredness or worries about not being able to keep up daily activities, job or household chores. The closer to a score of 100, the more improvement was made to the quality of life concerns of the PLWH.

There were increases from the time one mean score (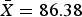) at times three and four with effect sizes of 0.61 and 0.29, indicating practical significance at these two measurements after the intervention. The four questions on the LS subscale with a pre-intervention time one score (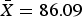) include such aspects as enjoyed living, felt in control, satisfied with social activity and pleased with health. These, although not statistically significant, increased by effect sizes of 0.48, 0.32 and 0.28 between times one and two, one and four and one and five respectively, indicating practical significance. The subscale about specific HW, such as cannot live the way I want to, worried about CD4 or viral load and about going to die, showed a statistically significant (*p* = .01) improvement after the intervention and through to time five.

FW were the more concerning result on the HAT-QoL for the 18 PLWH. Even though there was strong practical significance in the improvement after the intervention, the time one to five scores (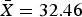, 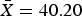, 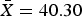, 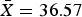 and 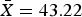) were far below the ideal score of 100. The effect sizes were 1.60, 1.62, 0.85 and 2.23 between time one and the other timelines. This subscale include worries about the lack of a fixed income, how to pay bills and not being able to care for self.

The scores on the scale for MW were close to 100 even before the intervention. The scores improved even more after the intervention from time two onwards, indicated practical significance and had effect sizes of 0.42 between times one and two, 0.73 between times one and three, 0.40 between times one and four and 0.62 between times one and five.

There was a statistical significant (*p* = .02) improvement in the DW of PLWH after the intervention. From time one (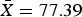) through to time five where the score reached almost 90 (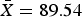) and a high score (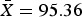) at time three. The subscale for worries about the doctor (physical therapist) included such aspects as accessibility, being included in the doctor's decision-making and a feeling that the doctor cares. These scores showed practical significant changes showing effect sizes of 0.43 between times one and three and 0.51 between times one and four. On the other hand, though it showed negative effect sizes between times one and two and times one and five which meant that times three and four scores of improvement did not stick. The SF subscale mean score for time one (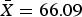) is fairly low in comparison with the other scales except for financial worries. The comprehensive community-based HIV stigma reduction intervention clearly made an impact and brought improvement in the SF worries of PLWH. The effect sizes of 1.94, 2.93, 3.99 and 3.05 indicate strong practical significant changes between time one and each of times two, three, four and five. The 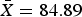 at time five is also much improved from the 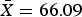 at time one and much closer to the ideal of 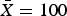.

The quality of life scale indicated statistical significant improvement on health and DW and practical significant strides in all but the worries about the physician which unfortunately pull down the total mean score at time five. But the post intervention measures show a steady increase in the all the other mean scores.

The research question as to whether the comprehensive community-based HIV stigma reduction intervention would make a difference to the HIV stigma experiences and health behaviour of PLWH has been answered satisfactorily. It would not be impossible to assume that we also saw a Hawthorne effect. Researchers took a keen interest in the participants, worked with them and sustained a presence within the community for at least a year and worked without a control group. Having a control group experiencing HIV stigma and not being offered any HIV stigma reduction did not seem a good option. The research team therefore accept that all the utilized instruments indicated either statistical or practical significance in the improvement of the HIV stigma experiences of PLWH and their health behaviour and ascribe the reduction in HIV stigma to the intervention.

## Conclusion

It can be concluded, based on the findings in this study, that there was no significant difference between the urban–rural findings on HIV stigma experiences and health behaviour of PLWH over time. This could have been due to the participants being all black Setswana-speaking South Africans from the North-West Province. The urban–rural data were therefore pooled for analysis. It should further be noted that the PLWH in this study had to be HIV positive for at least six months prior to the intervention and the purposive sampling suggested that they were already known participants in the health system. The comprehensive community-based intervention aimed at measuring and reducing HIV stigma for this group. It can be concluded that the intervention successfully changed aspects of their health behaviour, reduced their HIV stigma experiences, reduced the intensity of their HIV signs and symptoms and improved their quality of life. It was further found that the tenets of the intervention facilitated focused reflection by PLWH on HIV stigma experiences, health behaviour, HIV signs and symptoms and quality of life. For example, sharing knowledge on HIV stigma and how to cope with it (first tenet) afforded them an opportunity to openly discuss aspects of health behaviour such as reflection on responsible disclosure management, missing dosages of medicine, visits to a clinic and utilization of support. The second tenet aimed at equality in relationships among those with and those without HIV. It was found that the PLWH and PLC to them embraced the small-group interaction, openness and togetherness. This led to empowerment (third tenet) where PLWH and the PLC to them successfully worked together in self-initiated projects towards the reduction of HIV stigma in their communities.

Based on the findings in the demographic questionnaire and ACTG scale, it can be concluded that the 18 PLWH were a middle-aged group with minimal post-school education, a general lack of employment and part of the lower socio-economic population. Their participation in health care as measured directly after the intervention seemed satisfactory. They understood the value of being tested for HIV and the link between their CD4 count and treatment as well as the importance of not missing dosages of medication and utilizing health-care services when needed.

From further findings on the ACTG scale, it can be concluded that 2 of the 18 PLWH reflected behaviour scores indicative of risky health behaviour while eleven had perfect scores of nine. The reason mostly offered (five times) for missing dosages of medication was difficulty to take it at a specified time. This could be due to stigma and a lack of privacy at the specific time for taking medication that might disclose their HIV status.

The findings, on the HASI-P, show a decrease in the stigma experiences of PLWH on the five measured dimensions of VA, NSP, HCN, SI and FC. This is a statistical significant change-over-time. It can be concluded that the comprehensive community-based HIV stigma reduction intervention for PLWH and the PLC to them, was successful.

Findings on the scale measuring the intensity and frequency of the HIV signs and symptoms experienced by PLWH indicated statistical significant decrease on five of the nine subscales and practical significance on the other four. It can be concluded that the intensity and frequency of signs and symptoms declined after the intervention over time. This could be due to PLWH being able to share their experiences and put less focus on it because of the normalization thereof among the group.

Based on the findings that scores on all the subscales for the quality of life concerns of PLWH, except the total score and that of worries about the physician, increased, it can be concluded that the quality of life concerns of PLWH improved over time after the intervention. It mostly was a practical significant change, but the health and DW subscales showed a statistically significant improvement.

An overall conclusion, based on the mentioned findings, can be drawn that the comprehensive community-based HIV stigma reduction intervention was successful and potentially life-changing eventuality for PLWH. The measurement stretched over a year and while participants were not remunerated, their remaining in the process speaks of an ongoing engagement with HIV stigma, stigma reduction and health behaviour modification. The findings suggest that when HIV stigma reduces for PLWH, a conscious change in self-judgment and stigma experiences follow and this then leads to health behaviour change, less intense signs and symptoms, better adherence to treatment, responsible disclosure management, improved relationships and improved quality of life.

## Limitations of the study

The study is based on a comprehensive therapeutic intervention which limits the number of participants as it entails structured interactive processes and requires paired facilitators willing to share personal information about their HIV status. A different type of limitation concerns the specified six categories of PLC to the PLWH. This might not always be practical since PLWH might not have a partner, child, family member, friend, spiritual leader or community member to include.

## Recommendations

The simultaneous focus on HIV stigma experiences, physical experiences (signs and symptoms), behaviour matters (treatment adherence and disclosure of HIV status) and quality of life concerns, over a full year, is indicative of the comprehensiveness and therapeutic nature of the intervention used in this study. While the sample was rather small, it is recommended that this comprehensive community-based HIV stigma reduction intervention be repeated and further tested in larger samples. The findings may be indicative of a beneficial effect of the intervention where participants were faced with sensitive decisions. These could include the disclosure of their HIV status, the sharing of painful stigma experiences and the building, or rebuilding of important relationships. It could, for future testing of the intervention, be helpful to allow PLWH freedom to nominate any PLC of their choice rather than fitting into one of six designated categories. Another recommendation would be to add a further activity six months after the current study. This would need to focus on aspects of positive health behaviour coupled with psychosocial and spiritual well-being and an improved quality of life for PLWH.
